# Tolerability and Pharmacokinetic Evaluation of Inhaled Dry Powder Tobramycin Free Base in Non-Cystic Fibrosis Bronchiectasis Patients

**DOI:** 10.1371/journal.pone.0149768

**Published:** 2016-03-09

**Authors:** Marcel Hoppentocht, Onno W. Akkerman, Paul Hagedoorn, Jan-Willem C. Alffenaar, Tjip S. van der Werf, Huib A. M. Kerstjens, Henderik W. Frijlink, Anne H. de Boer

**Affiliations:** 1 Department of Pharmaceutical Technology and Biopharmacy, University of Groningen, Groningen, the Netherlands; 2 Department of Pulmonary Diseases and Tuberculosis, University of Groningen, University Medical Center Groningen, Groningen, the Netherlands; 3 Department of Clinical Pharmacy and Pharmacology, University of Groningen, University Medical Center Groningen, Groningen, the Netherlands; 4 Department of Internal Medicine, University of Groningen, University Medical Center Groningen, Groningen, the Netherlands; University of Ottawa, CANADA

## Abstract

**Rationale:**

Bronchiectasis is a condition characterised by dilated and thick-walled bronchi. The presence of *Pseudomonas aeruginosa* in bronchiectasis is associated with a higher hospitalisation frequency and a reduced quality of life, requiring frequent and adequate treatment with antibiotics.

**Objectives:**

To assess local tolerability and the pharmacokinetic parameters of inhaled excipient free dry powder tobramycin as free base administered with the Cyclops dry powder inhaler to participants with non-cystic fibrosis bronchiectasis. The free base and absence of excipients reduces the inhaled powder dose.

**Methods:**

Eight participants in the study were trained in handling the device and inhaling correctly. During drug administration the inspiratory flow curve was recorded. Local tolerability was assessed by spirometry and recording adverse events. Serum samples were collected before, and 15, 30, 45, 60, 75, 90, 105, 120 min; 4, 8 and 12 h after inhalation.

**Results and Discussion:**

Dry powder tobramycin base was well tolerated and mild tobramycin-related cough was reported only once. A good drug dose-serum concentration correlation was obtained. Relatively small inhaled volumes were computed from the recorded flow curves, resulting in presumably substantial deposition in the central airways—i.e., at the site of infection.

**Conclusions:**

In this first study of inhaled dry powder tobramycin free base in non-cystic fibrosis bronchiectasis patients, the free base of tobramycin and the administration with the Cyclops dry powder device were well tolerated. Our data support further clinical studies to evaluate safety and efficacy of this compound in this population.

## Introduction

Bronchiectasis is a persistent and frequently progressive condition characterised by dilated and thick-walled bronchi. This pathology can result from many underlying conditions, including post-infectious conditions. It is often divided in bronchiectasis caused by cystic fibrosis (CF) and non-cystic fibrosis (non-CF) bronchiectasis. The main symptoms of bronchiectasis are cough and chronic sputum production [1]. The infectious burden stimulates neutrophilic and inflammatory mediator responses in the airways [2]. Ongoing structural damage has been referred to as the vicious circle in bronchiectasis [3]. Different studies show that *Haemophilus influenzae* was isolated in 29–42% and *Pseudomonas aeruginosa* (*Psa*) in 13–31% of the patients with stable non-CF bronchiectasis [4,5]. The presence of *Psa* in patients with bronchiectasis is associated with increased sputum production, more extensive bronchiectasis on high-resolution computed tomography (HR-CT) of the chest, a higher hospitalisation frequency, and a reduced quality of life [2,6–9].

Current treatment practice for non-CF bronchiectasis patients chronically infected with *Psa* consists of pulmonary tobramycin (as sulphate) or colistin (as sulphomethate sodium) often in combination with orally administered macrolides [1,10]. However, the BTS guidelines are still reticent about using macrolides in this population. Both inhaled drugs are most frequently administered by wet nebulisation. Nebulised tobramycin sulphate is usually administered for 28 days in 2 daily doses of 300 mg each, followed by 28 days without tobramycin therapy to reduce the risk of side effects and antibiotic resistance. This regimen was originally tested in patients with CF [11–13]; trials conducted with inhaled tobramycin in non-CF bronchiectasis patients with chronic *Psa* have shown clinical improvement and a reduction in bacterial density too [14]. An alternative to wet nebulisation of tobramycin sulphate is the TOBI^®^ Podhaler^™^. Tobramycin sulphate inhalation powder (TIP), administered with the Podhaler^™^ to CF patients that are chronically infected with *Psa* appeared to be safe and effective [15]. Pharmacokinetic parameters and efficacy of a 112 mg TIP dose twice daily were similar to 300 mg nebulised tobramycin sulphate solution twice daily [15]. However, the re-usable capsule based dry powder inhaler (DPI) and voluminous powder formulation of the sulphate containing various excipients have some disadvantages, notably, the large number of steps to administer one dose [16]. No clinical studies with dry powder tobramycin have been carried out in non-CF bronchiectasis patients to date.

The aim of this study was to assess local tolerability and the pharmacokinetic parameters of increasing doses of dry powder tobramycin free base administered using the Cyclops DPI without excipients to participants with non-CF bronchiectasis.

## Methods

### Materials

Tobramycin free base was obtained from Spruyt Hillen BV (the Netherlands) and spray dried at the Department of Clinical Pharmacy and Pharmacology of the University Medical Center Groningen (UMCG) following previously described procedures [17]. The free base of tobramycin was chosen instead of the commonly used sulphate salt based on its favourable physico-chemical properties and the sulphate group increases the amount of powder to be inhaled [17]. The Cyclops DPIs used during this study were also described earlier [17].

### Participants

Eight participants with non-CF bronchiectasis, confirmed by HR-CT, were recruited in the outpatient department of the Department of Pulmonary Diseases and Tuberculosis of the UMCG. The baseline characteristics of the participants are listed in Table 1. The criteria for exclusion were partly based on the contra-indications and known drug-drug interactions of the TOBI^®^ Podhaler^™^ [18]. In- and exclusion criteria are listed in Table 2. Written informed consent was obtained from all participants.

**Table 1:** Participant characteristics.

Participant	Sex	Age	FEV_1_ /FVC	FEV1 Predicted (%)	BMI	Asthma
P1	F	60	67	113	43	Yes
P2	F	68	86	74	32	No
P3	F	69	46	31	31	Yes
P4	M	69	65	71	23	No
P5	F	64	71	71	25	No
P6	F	63	77	106	23	No
P7	F	57	71	92	39	Yes
P8	F	73	61	82	29	Yes

**Table 2:** In- and exclusion criteria.

**Inclusion criteria**:
Age 18 years or older
Obtained informed consent
Patients having bronchiectasis (confirmed with HR-CT of the chest)
**Exclusion criteria**:
Patients with cystic fibrosis
Pregnant or breast feeding
Subjects with known or suspected renal, auditory, vestibular or neuromuscular dysfunction, or with severe, active haemoptysis
History of adverse events on previous tobramycin or other aminoglycoside use
Concurrent use of cyclosporin, cisplatin, amfotericin B, cephalosporins, polymyxins, vancomycin or NSAIDs

### Study objectives and design

**Fig 1:**
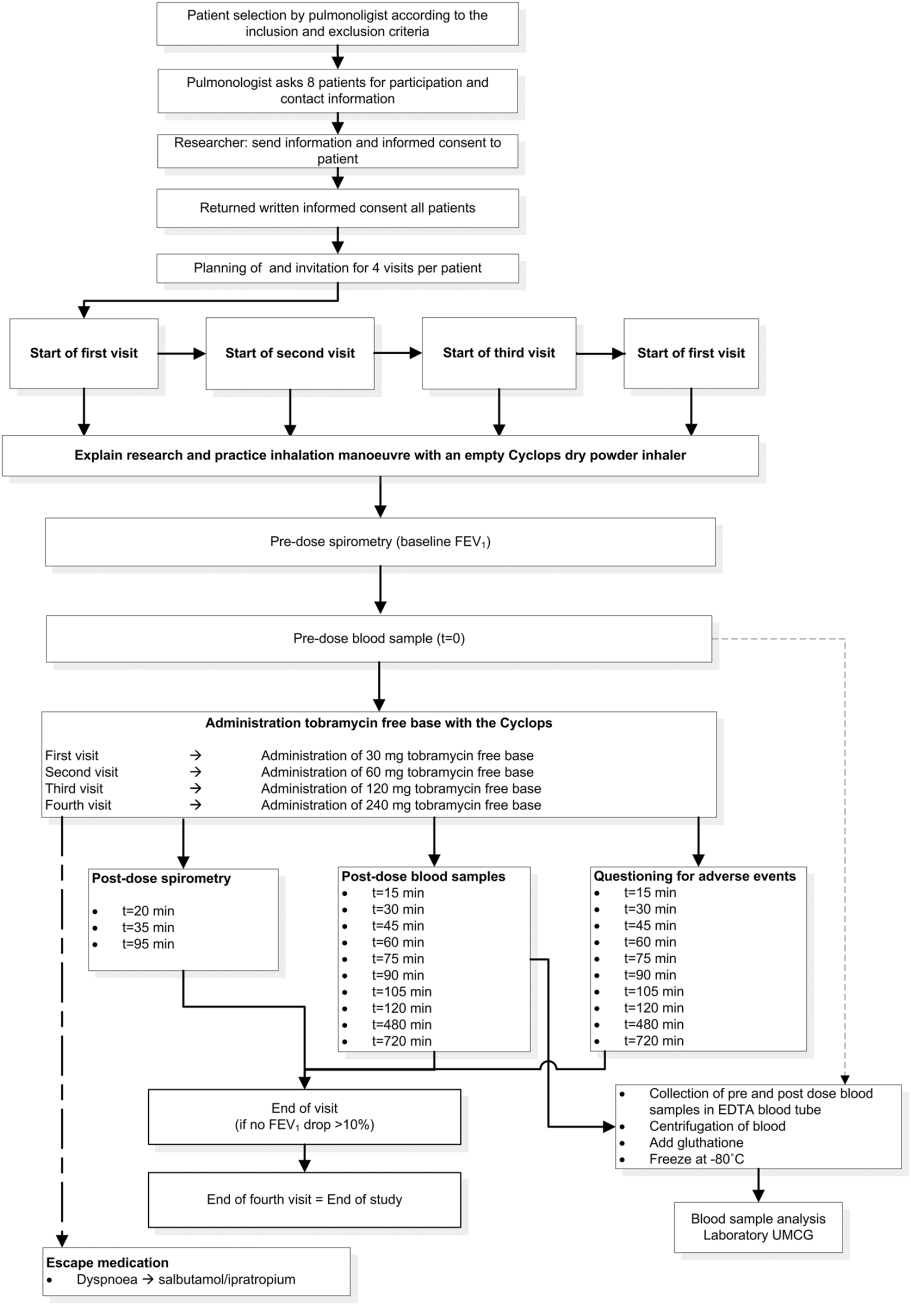
CONSORT flow diagram tobra-02 study.

The primary objectives were to assess both local tolerability and pharmacokinetics of dry powder tobramycin free base administered using the Cyclops in the target population. During four consecutive visits, at least 7 days apart, the participants received a 30, 60, 120 or 240 mg dose of dry powder tobramycin from the Cyclops. Each blister contained 30 mg of tobramycin; the higher doses were administered in multiple successive blisters and inhalers. This study was performed as single centre, dose-escalation study at UMCG location Beatrixoord (Haren, the Netherlands). The study flowchart is depicted in Fig 1.

### Tolerability

Local tolerability was assessed by spirometry, combined with active questioning and passive monitoring by recording remarks about adverse events made by the participants. Spirometry was performed before (S0) inhalation and 20 (S1), 35 (S2) and 95 (S3) minutes after inhalation. A drop in FEV_1_ of 10% or more compared to baseline FEV_1_ (S0) was considered significant. Active questioning for adverse events was done every time a blood sample was drawn. Furthermore, before inhalation the creatinine level of every participant was measured as baseline to check for decreased kidney function. The creatinine clearance was calculated using the Cockroft Gault formula.

### Serum sampling and analysis

Blood samples were collected before pulmonary administration of the study drug (t = 0), and 15, 30, 45, 60, 75, 90, 105, 120 min; 4, 8 and 12 hours after inhalation. The samples were centrifuged for 5 min at 3000 rpm and subsequently stored at -80°C until analysis. Tobramycin serum concentrations were analysed using a modified immunoassay method Syva^®^ *Emit*^*®*^ *2000* Tobramycin Assay (Siemens Healthcare, Germany) combined with the ARCHITECT c8000 (Abbott Diagnostics, U.S.A.) [19].

### Pharmacokinetic analysis

The area under the concentration time curve from t = 0 to t = 12 h (AUC_0-12_) was calculated by the log-lineartrapezoidal rule using the KinFIT function in the pharmacokinetic software package MW/Pharm (Mediware, the Netherlands) [20]. The maximum serum concentration (C_max_) and time to maximum serum concentration (t_max_) were derived from the concentration-time curves. The delivered dose was computed from weighed dose and inhaler residue determined by gravimetric analysis for the first two participants and by chemical and gravimetrical analysis for the others. Gravimetrical analysis was performed immediately after inhalation and chemical analysis on the same day of administration. We used a 2,4,6-Trinitrobenzene Sulfonic Acid (TNBSA) assay to chemically quantify the amount of tobramycin retained in the Cyclops DPIs [17].

### Recording of the inspiratory flow curve

Prior to inhalation of the study drug, study participants received inhalation instructions followed by training regarding handling of the device and performing a correct inhalation manoeuvre. Training was done using an empty Cyclops connected to a laptop, with self-written software (LabVIEW, National Instruments, the Netherlands) for recording and processing of flow curves generated through the device. A differential pressure gauge (Sitrans P250, Siemens, Germany) was used to measure the pressure drops generated across the inhaler, after prior pressure drop versus flow rate calibration with a thermal mass flow meter (Brooks Smart Mass Flow Meter 5863S, USA). Inhaler instrumentation was performed without changing the inhaler resistance or interfering with the aerosol delivery [17]. First when a series of consistent flow curves meeting the criteria for good inhaler performance was obtained during training, a similarly instrumented Cyclops with tobramycin was handed to the participant. Also during the drug administration the inspiratory flow-rate was recorded to be able to explain unexpected pharmacokinetic results, and to ascertain that the participants generated a 4 kPa pressure drop—corresponding with the target flow rate of 34 L/min [17].

### Ethics

The study protocol (S2 Text) was approved by the medical ethical review committee (METc) of the UMCG (METc number 2013.024) on May 15^th^ 2013 and was performed according to the Helsinki declaration. The study was registered at www.clinicaltrials.gov (NCT02035488). The authors confirm that all ongoing and related trials for this drug/intervention are registered. However, the study was registered after patient recruitment began due to miscommunication. After notification, this issue was resolved. The study lasted from July 1^st^ 2013 (start patient inclusion) until June 13^th^ 2014 (last day of the study).

## Results

### Participants

Eight participants were enrolled and all completed the study.

### Inhalation manoeuvres

Training of respiratory manoeuvres was successful in all participants. All were also able to hold their breath for 10 sec after inhalation of the drug to facilitate deposition by sedimentation in the airways.

### Local tolerability

Administration of dry powder tobramycin free base using the Cyclops was well tolerated. Table 3 shows that four participants showed significant drops in FEV_1_ (≥ 10%) at some time point after dose administration. In total six significant drops were recorded out of 32 measurements (19%), 4 times after a low dose (30–60 mg) and 2 times after a high dose (120–240 mg). The first two participants had slight complaints of a bad taste after inhalation of the first dose (30 mg). For this reason, the participants were advised to rinse their mouth with water after the complete dose was administered. Thereafter, none of the participants reported this adverse event. Two participants reported mild cough—one after a dose of 240 mg, 7 hours after inhalation; the other reported cough after active questioning after a dose of 30 mg, 1 hour after inhalation.

**Table 3:** Drops in FEV_1_ >10% during all four visits.

Participant	Visit 1 (30 mg)	Visit 2 (60 mg)	Visit 3 (120 mg)	Visit 4 (240 mg)
P1	No	No	No	No
P2	No	No	Yes (S1: 18%; S2: 11%)	No
P3	Yes (S1: 14%; S2: 10%)	Yes (S3: 10%)	No	No
P4	No	No	No	No
P5	No	No	No	No
P6	No	No	No	No
P7	Yes (S1: 13%; S3: 12%)	No	No	Yes (S1: 10%; S2: 10%)
P8	Yes (S3: 10%)	No	No	No

S indicates during which of the 3 spirometry measurements after inhalation the drop occurred.

### Pharmacokinetic analysis

**Fig 2:**
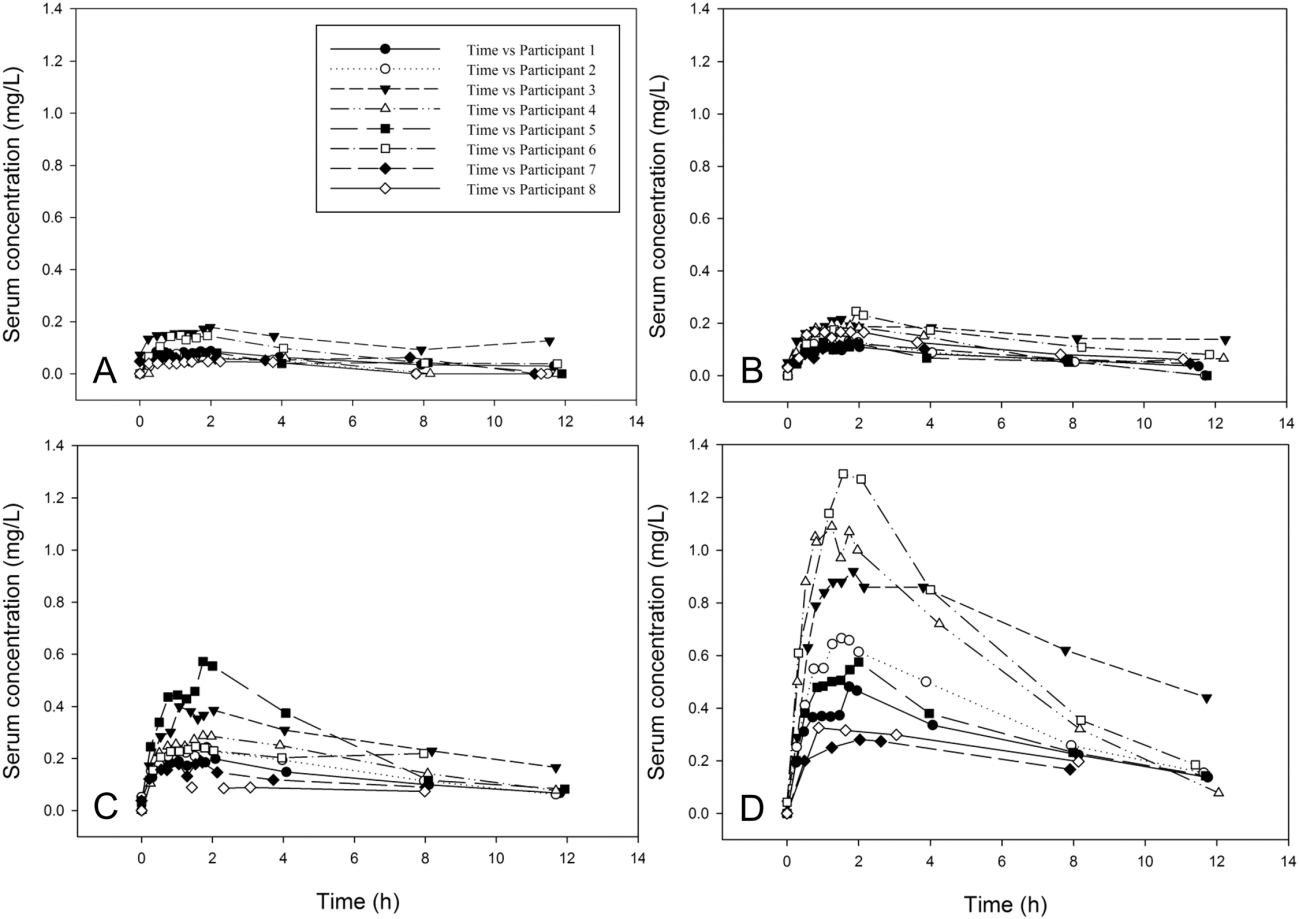
Individual serum concentrations of tobramycin following administration of a 30 (A), 60 (B), 120 (C) or 240 (D) mg dry powder tobramycin dose from the Cyclops.

**Fig 3:**
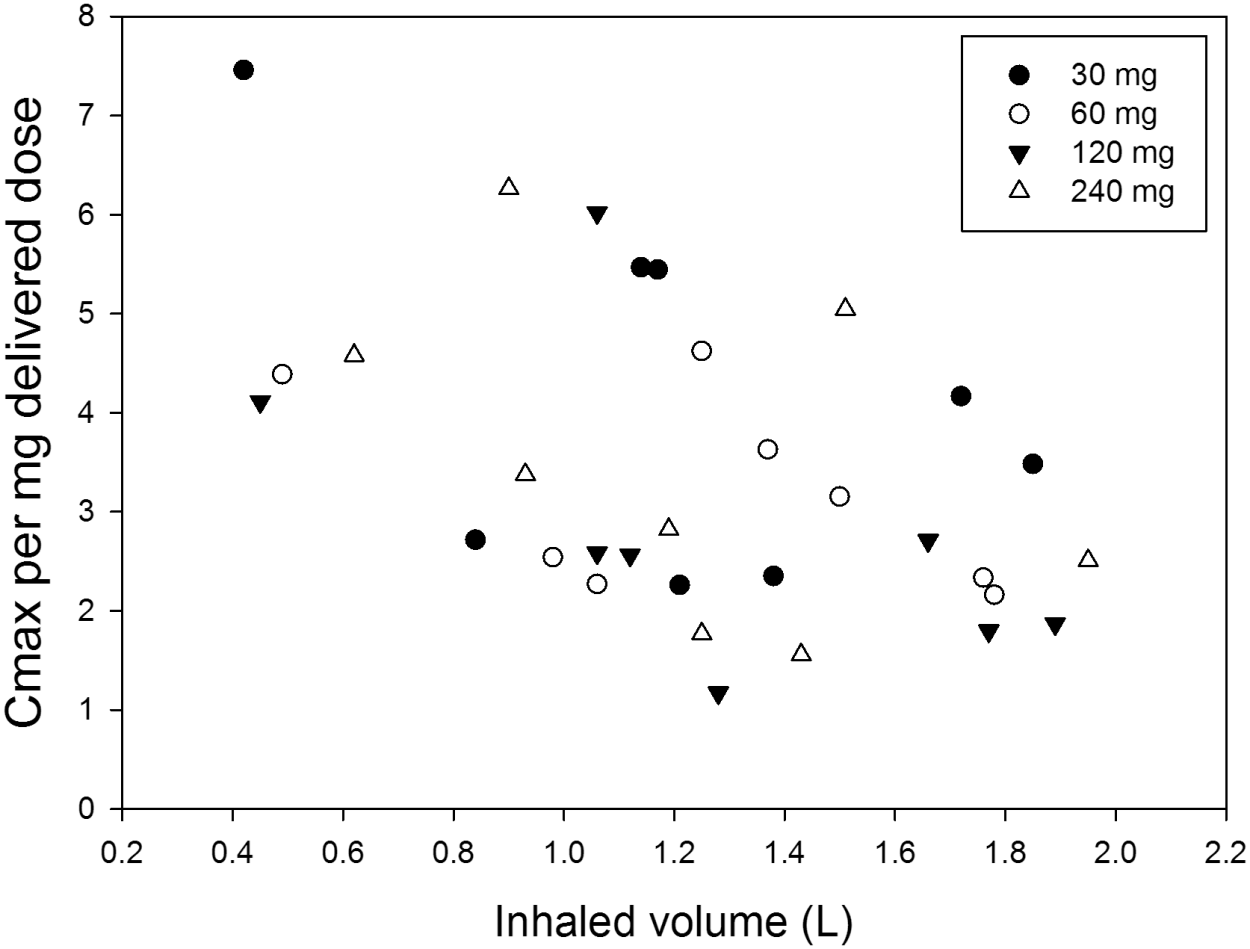
The C_max_ per milligram delivered dose as function of the inhaled volume.

All mean pharmacokinetic parameters investigated are summarised in Table 4. As expected, the mean C_max_ and mean AUC_0-12_ rose approximately by two-fold after each doubling of the dose. The t_max_ was the same, 1.6 (± 0.08) h, for all four doses investigated. Fig 2 shows the serum concentration-time curves of the individual participants after 30 (Fig 2A), 60 (Fig 2B), 120 (Fig 2C) and 240 mg (Fig 2D) dry powder tobramycin. Some data points are missing due to failed blood draws. Therefore, the mean AUC_0-12_ presented in Table 4 was calculated for 6 participants. Apart from inter-individual differences (Fig 2), also large intra-individual differences were observed in some participants. For example, participant 5 showed a C_max_ of 0.57 mg/L after a 120 mg dose (delivered dose 95 mg), but a 240 mg dose (delivered dose 204 mg) resulted in a C_max_ of only 0.58 mg/L. Fig 3 shows the C_max_ per mg delivered dose as function of the inhaled volume; the figure indicates a strong trend for increasing normalised C_max_ with decreasing inhaled volume.

**Table 4:** Pharmacokinetic parameters (mean ± standard deviation).

Parameters	Visit 1(30 mg)	Visit 2(60 mg)	Visit 3(120 mg)	Visit 4(240 mg)
Delivered dose(mg)	23 ± 4.8	53 ± 2.3	97 ± 9.7	198 ± 11.9
AUC_0-12_ (h mg/L)	0.40 ± 0.72	1.03 ± 0.56	2.26 ± 0.77	5.36 ± 2.10
C_max_ (μg/L)	105 ± 45	173 ± 48	277 ± 148	703 ± 365
t_max_ (h)	1.57 ± 0.48	1.45 ± 0.41	1.64 ± 0.31	1.60 ± 0.39

The area under the curve from t = 0 to t = 12 h (AUC_0-12_) was calculated using MW/Pharm. The maximum serum concentration (C_max_) and time to maximum serum concentration (t_max_) were derived from the concentration-time curves. It was particularly difficult to obtain blood from participants 7 and 8 during visit 3 and 4.

## Discussion

In this study we assessed the local tolerability and pharmacokinetic parameters of escalating doses of dry powder tobramycin free base using the Cyclops in participants with non-CF bronchiectasis. We demonstrated that inhalation of dry powder tobramycin base from the Cyclops is well tolerated.

Coughing is often reported as an adverse event immediately after inhalation of tobramycin, either by wet nebulisation or dry powder inhalation, both in CF and non-CF bronchiectasis patients [21–23]. In this study, only two participants started coughing after inhalation, each during only one out of four visits. One participant reported cough 7 hours after inhalation, making causality of dry powder tobramycin less likely. We believe that the high inhaler resistance to airflow and excellent powder dispersion by the Cyclops may explain the very low frequency of coughing. The tobramycin particles, of which almost 90% is between 1 and 5 μm, enter the respiratory tract at a flow rate of only 34 L/min [17]. This combination of beneficial features prevents the deposition of substantial drug fractions in the oropharynx, which is the common trigger for coughing. Based on the experience with colistin sulphomethate and colistin sulphate [24], where using the sulphomethate salt reduced cough compared to using the sulphate salt, we speculate that the use of tobramycin free base instead of the sulphate salt might also help to reduce cough. In addition, the lower powder dose to be inhaled for the free base (65.6% compared to the sulphate) without excipients may also have contributed to reduced cough. Rinsing the mouth with water after administration of the full dose solved the reported bad taste of the two participants after their first visit. The bad taste is known from nebulised tobramycin sulphate.

In four participants a drop in FEV_1_ of 10% or more was observed during one or two visits. These four participants were diagnosed with asthma, all of the drops were without complaints of dyspnoea. In half of the cases the drop in FEV_1_ was exactly 10%. No correlation was found between the drops in FEV_1_ and the different time points of spirometry nor with the dose administered. All drops in FEV_1_ observed during the first two measurements (S1 and S2), were spontaneously reversed without the use of bronchodilators. In a previous study with nebulised tobramycin 3 out of 26 participants showed a drop in FEV_1_ > 10%, but also 5 out of 27 participants in the placebo group showed a drop in FEV_1_ > 10%. They considered a drop in FEV_1_ of 10% not to be an adverse event to inhaled tobramycin [25]. Others suggest that respiratory adverse events are more common in non-CF bronchiectasis patients than in CF patients. They state that this is probably caused by underlying morbidities like asthma, the greater age of these patients, and a greater history of smoking [14,23,26]. The clinical relevance needs to be determined in larger phase 2 and 3 studies.

The computed normalised C_max_ values (C_max_ per mg delivered dose) in our study are in a wide range between 1.17 and 7.46 μg/L per mg of delivered dose, with an overall average of 3.41 μg/L. The delivered doses were derived from the inhaler retentions measured. In a study in healthy volunteers, the Podhaler^™^ 80 mg dose, after correction for the inhaler losses, the sulphate group and the excipients, yielded a normalised C_max_ value of 9.57 μg/L per mg delivered free base [27]. In CF patients, normalised C_max_ values of approximately 22 μg/L per mg delivered free base could be derived (more or less independent of the dose), assuming that Podhaler^™^ losses were similar to earlier reports [15]. The wide range of normalised C_max_ values from the Cyclops in our study and the lower C_max_ value compared to studies in CF patients and healthy volunteers with the Podhaler^™^ are remarkable. Further investigation is needed to elucidate whether they result from a difference in inhaler performance, or from differences in the study populations—or both.

The Cyclops delivered doses derived from inhaler residues were quite consistent and are on average (all doses, all patients) 82.1% of the doses weighed into the blisters (RSD = 12.2%). Therefore, delivered dose variation does not seem to explain the wide range of normalised C_max_ values in this study. In a previous study good dispersion performance of the Cyclops was already demonstrated [17]; fairly consistently delivered fine particle fractions (FPF < 5 μm) of approximately 75% of the weighed doses were computed. Losses in the oropharynx between the Cyclops and the Podhaler^™^ may have been different due to a difference in inhaler mouthpiece design, although the exit velocity at 35 L/min from the Cyclops (24.3 m/s in our study) is the same as that from the Podhaler^™^ at 80 L/min (24.7 m/s) in the studies with this device. Nevertheless, aerosol plume geometry and jet effects resulting in return flows in the oral cavity, may be different and greater for the Cyclops compared to the Podhaler^™^ in spite of comparable exit velocities. Beyond the oral cavity however, at a distance from the mouthpiece, the more than two times lower flow rate from the Cyclops at the same pressure drop must result in lower inertial deposition in the first bifurcations. Since almost no tobramycin related cough was reported or observed during this study, it is safe to assume that indeed no large losses in the oropharynx from the Cyclops occurred. Therefore, a difference in results from the different studies seems most likely the result of the inhalation manoeuvre or a difference in disease related aspects, between the subject populations. In our study, relatively small inhaled volumes ranging from only 0.42 to 1.95 L were computed from the flow curves recorded during drug administration. They were less than 50% of recorded Vital Capacities, in spite of the instructions given to inhale as deep as possible, and cannot be explained by dyspnoea since all participants were able to comply with the recommended breath-hold pause of at least 10 s after inhalation. These low volumes must have resulted in substantial deposition in the upper and central respiratory tract and only marginal aerosol penetration into the most distal airways, where absorption is supposed to be fastest (resulting in a high C_max_) [28]. However, the most distal airways may not be the most relevant target area in non-CF bronchiectasis patients, since it is known that bacterial infections in this population are mainly located in the bronchi and less in the bronchioles and alveoli [29]. Surprisingly, a strong trend was found for increasing normalised C_max_ with decreasing inhaled volume (Fig 3). Comparison with the Podhaler^™^ studies in this respect is not possible, as flow curves during drug administration were not recorded in the studies performed with this device. With the Podhaler^™^, almost 40% of the whole lung dose was recovered from the peripheral airways, which suggests that inhaled volumes were considerably higher, presumably causing the higher C_max_ values.

The finding of increased normalised C_max_ with decreasing inhaled volume was unexpected, and clearly needs further clinical investigations. In patients with bronchiectasis the bronchial circulation can be increased from 1% to as much as 30% of the cardiac output due to increased inflammation [30,31]. It can be hypothesised that the higher blood circulation increases the absorption rate as drugs like tobramycin and other antibiotics penetrate faster also in opposite direction from the systemic circulation into lung tissue in patients with pulmonary infections like pneumonia [32,33]. Because non-CF bronchiectasis is a progressively deteriorating condition accompanied by increased inflammation, it could be that the C_max_ changes with the degree of inflammation. This could also explain why in previous studies with the TOBI^®^ Podhaler^™^ normalised C_max_ values were much higher for CF patients compared to healthy volunteers [15,27]. These aspects remain unclear from all deposition studies however, and should be addressed in future clinical investigations with inhaled antibiotics.

Our data are limited to AUC, t_max_ and C_max_ results in serum; the topical tobramycin concentrations in the airways—i.e. at the site of infection—were not measured. A phase 2 study evaluating safety and efficacy in non-CF bronchiectasis patients should be performed next. Based on the current data, we recommend 120 and 240 mg dry powder tobramycin doses by the Cyclops.

## Conclusions

This is the first pilot study describing the use of dry powder tobramycin free base in non-CF bronchiectasis patients. The free base was well tolerated and this positive result invites for further clinical studies with the Cyclops dry powder inhaler to evaluate safety and efficacy of this compound in non-CF bronchiectasis patients.

## Supporting Information

S1 Text TREND Statement Checklist. (PDF)

S2 Text Study Protocol Tobra-02. (PDF)

## Abbreviations

CF: Cystic fibrosis
DPI: Dry powder inhaler
HR-CT: High-resolution computed tomography
*Psa*: *Pseudomonas aeruginosa*
TIP: Tobramycin inhalation powder
