# Supplementary material for: Tolerability and Pharmacokinetic Evaluation of Inhaled Dry Powder Tobramycin Free Base in Non-Cystic Fibrosis Bronchiectasis Patients
Source: PLoS One. 2016 Mar 9;11(3):e0149768. doi: 10.1371/journal.pone.0149768 (PMC4784940; doi:10.1371/journal.pone.0149768)
Supplement: S2 Text — (PDF) [file pone.0149768.s002.pdf]

Pharmacokinetic evaluation and tolerability of dry powder tobramycin by a novel device in patients with non cystic fibrosis bronchiectasis

'Pharmacokinetic evaluation and tolerability of dry powder tobramycin by a novel device in patients with non cystic fibrosis bronchiectasis'

**Protocol ID**	**Pharmacokinetic evaluation and tolerability of dry powder tobramycin by a novel device in patients with non cystic fibrosis bronchiectasis**
**Short title**	**Tobra-02 study**
**EudraCT number**	**2012-004437-16**
**Version**	**2**
**Date**	**14-04-2013**
**Coordinating investigator/project leader**	***Prof. Dr. H.A.M. Kerstjens***<br>***Contact data:***<br>***Head Dept of Pulmonary Diseases and Tuberculosis***<br>***University Medical Center Groningen***<br>***Post box 30.001, 9700RB Groningen, NL***<br>***h.a.m.kerstjens@umcg.nl***<br>***tel. +31 50 36 10280***
**Principal investigator(s) (in Dutch: hoofdonderzoeker/ uitvoerder)**	***O.W. Akkerman***<br>***Contact data:***<br>***Dept of Pulmonary Diseases and Tuberculosis***<br>***University Medical Center Groningen***<br>***Post box 30.001, 9700RB Groningen, NL***<br>***o.w.akkerman@umcg.nl***<br>***Tel. + 31 50 36 10196***
**Sponsor (in Dutch: verrichter/opdrachtgever)**	***University Medical Center Groningen, Department of Pulmonary Disease and Tuberculosis, and RijksUniversiteit Groningen, Pharmaceutical Technology and Biopharmacy, RUG.***

**Subsidising party**	
**Independent physician(s)**	***Dr. N.H.T. ten Hacken, pulmonary physician UMCG***<br>***Contact data:***<br>***Dept of Pulmonary Diseases and Tuberculosis***<br>***University Medical Center Groningen***<br>***Post box 30.001, 9700RB Groningen, NL***<br>**n.h.t.ten.hacken@umcg.nl**<br>***tel. +31 50 36 14574***
**Laboratory sites**	***University Medical Center Groningen***
**Pharmacy**	***University Medical Center Groningen***

PROTOCOL SIGNATURE SHEET

Name	Signature	Date
For non-commercial research,<br>Head of Department:	Prof. Dr. H.A.M. Kerstjens	
Principal Investigator:	O.W. Akkerman	
Co-investigator:	M. Hoppentocht	
Co-investigator:	A. Lexmond	
Co-investigator:	Prof. dr. H.W. Frijlink	
Co-investigator:	dr. A.H. de Boer	
Co-investigator:	Prof. dr. T.S. van der Werf	
Co-investigator:	dr. J.W.C. Alffenaar	

TABLE OF CONTENTS

2. INTRODUCTION AND RATIONALE 11
3. OBJECTIVES 16
4. STUDY DESIGN 17
5. STUDY POPULATION 20
   5.1 Population (base) 20
   5.2 Inclusion criteria 20
   5.3 Exclusion criteria 20
   5.4 Sample size calculation 20
6. INVESTIGATIONAL PRODUCT 21
   6.1 Name and description of investigational product(s) 21
   6.2 Summary of findings from non-clinical studies 21
   6.3 Summary of findings from clinical studies 21
   6.4 Summary of known and potential risks and benefits 22
   6.5 Description and justification of route of administration and dosage 23
   6.6 Dosages, dosage modifications and method of administration 23
   6.7 Preparation and labelling of Investigational Medicinal Product 25
   6.8 Drug accountability 26
7. METHODS 27
   7.1 Study parameters/endpoints 27
      7.1.1 Main study parameter/endpoint 27
   7.2 Randomisation, blinding and treatment allocation 27
   7.3 Study procedures 27
   7.4 Withdrawal of individual subjects 29
      7.4.1 Specific criteria for withdrawal 29
   7.5 Replacement of individual subjects after withdrawal 29
   7.6 Follow-up of subjects withdrawn from treatment 29
   7.7 Premature termination of the study 29
8. SAFETY REPORTING 30
   8.1 Section 10 WMO event 30
   8.2 AEs, SAEs and SUSARs 30
      8.2.1 Adverse events (AEs) 30
      8.2.2 Serious adverse events (SAEs) 30
      8.2.3 Suspected unexpected serious adverse reactions (SUSARs) 31
   8.3 Annual safety report 32
   8.4 Follow-up of adverse events 32
   8.5 Data Safety Monitoring Board (DSMB) / Safety Committee 32
9. STATISTICAL ANALYSIS 33
   9.1 Primary study parameter(s) .... **Error! Bookmark not defined.**
10. ETHICAL CONSIDERATIONS 34
   10.1 Regulation statement 34
   10.2 Recruitment and consent 34

10.3 Benefits and risks assessment, group relatedness 34
10.4 Compensation for injury 34
10.5 Incentives 35
11. ADMINISTRATIVE ASPECTS, MONITORING AND PUBLICATION 36
11.1 Handling and storage of data and documents 36
11.2 Monitoring and Quality Assurance 36
11.3 Amendments 36
11.4 Annual progress report 36
11.5 End of study report 37
11.6 Public disclosure and publication policy 37
12. STRUCTURED RISK ANALYSIS 38
12.1 Potential issues of concern 38
12.2 Synthesis 41
13. REFERENCES 42

LIST OF ABBREVIATIONS AND RELEVANT DEFINITIONS

**ABR**	**ABR form, General Assessment and Registration form, is the application form that is required for submission to the accredited Ethics Committee (In Dutch, ABR = Algemene Beoordeling en Registratie)**
**ABPA**	**Allergic Bronchopulmonary Aspergillosis**
**AE**	**Adverse Event**
**AR**	**Adverse Reaction**
**AUC**	**Area Under the Curve**
**Cmax**	**Maximum Plasma Concentration**
**CA**	**Competent Authority**
**1. CCMO**	**Central Committee on Research Involving Human Subjects; in Dutch: Centrale Commissie Mensgebonden Onderzoek**
**CF**	**Cystic Fibrosis**
**CFU**	**Colony Forming Units**
**CHMP**	**Committee for Medicinal Products for Human Use**
**CL**	**Clearance**
**CV**	**Curriculum Vitae**
**DP**	**Dry Powder**
**DPI**	**Dry Powder Inhaler**
**DSMB**	**Data Safety Monitoring Board**
**ERS**	**European Respiratory Society**
**EU**	**European Union**
**EudraCT**	**European drug regulatory affairs Clinical Trials**
**F**	**Bioavailability**
**FEV1**	**Forced Expiratory Volume in one second**
**GCP**	**Good Clinical Practice**
**HR-CT**	**High Resolution Computed Tomography Scan**
**IB**	**Investigator’s Brochure**
**IC**	**Informed Consent**
**IMP**	**Investigational Medicinal Product**
**IMPD**	**Investigational Medicinal Product Dossier**
**Ka**	**absorption rate constant**
**METC**	**Medical research ethics committee (MREC); in Dutch: medisch ethische toetsing commissie (METC)**
**PVC**	**Polyvinyl Chloride**
**(S)AE**	**(Serious) Adverse Event**

**SPC**	**Summary of Product Characteristics (in Dutch: officiële productinfomatie IB1-tekst)**
**Sponsor**	**The sponsor is the party that commissions the organisation or performance of the research, for example a pharmaceutical company, academic hospital, scientific organisation or investigator. A party that provides funding for a study but does not commission it is not regarded as the sponsor, but referred to as a subsidising party.**
**SUSAR**	**Suspected Unexpected Serious Adverse Reaction**
**$T_{1/2\ el}$**	**Terminal elimination half-life**
**Tmax**	**Time to maximum plasma concentration**
**TIP**	**Tobramycin Inhalation Powder**
**TIS**	**Tobramycin Inhalation Solution**
**UMCG**	**University Medical Center Groningen**
**Wbp**	**Personal Data Protection Act (in Dutch: Wet Bescherming Persoonsgevens)**
**WMO**	**Medical Research Involving Human Subjects Act (in Dutch: Wet Medisch-wetenschappelijk Onderzoek met Mensen**

SUMMARY

Rationale: Bronchiectasis is a persistent and frequently progressive condition characterized by dilated and thick-walled bronchi retaining sputum. There are multiple underlying causes, of which cystic fibrosis (CF) is the best known, but frequently no cause can be identified. The main symptoms of bronchiectasis are cough and chronic sputum production. There is a state of constant colonization with bacteria, which frequently causes exacerbations. The presence of *Pseudomonas aeruginosa* is an unfavorable prognostic indicator and is associated with increased sputum production, more extensive bronchiectasis on HR-CT of the thorax, more hospitalizations and reduced quality of life. Until now, most patients with non-CF bronchiectasis who are colonized with *P. aeruginosa* receive inhaled tobramycin every other month, by use of a nebulizer. However, this delivery system has several disadvantages, like a low lung deposition and pollution with tobramycin in the surrounding environment. With an efficient dry powder inhaler (DPI), a three to six fold higher lung deposition compared to a nebulizer can be obtained. Therapy with a DPI is also less time consuming compared to nebulisation. Nebulised tobramycin is used most in routine care; there is also one, rather poorly characterized DPI for tobramycin available, though this DPI is not registrered for non-CF bronchiectasis. We will investigate dry powder tobramycin (DP tobramycin) in a novel device in patients with non-CF bronchiectasis colonized with *P. aeruginosa*.

Objective: The main objectives are to investigate the pharmacokinetic properties of DP tobramycin at different dosages together with the local tolerability of DP tobramycin via the Cyclops® at different dosages.

Study design: single center, single ascending, single dose, response study.

Study population: 8 patients with non-CF bronchiectasis

Main study parameters/endpoints:

The following pharmacokinetic parameters will be calculated: actual dose (dose minus remainder in inhaler after inhalation), AUC_{0-12} (area under the curve from 0 -12 h), C_{max} (maximum plasma concentration), T_{max} (time to maximum plasma concentration), Ka (absorption rate constant), $T_{1/2\ el}$ (terminal elimination half-life), CL/F (clearance following pulmonary administration (F= bioavailability)).

Local tolerability of DP tobramycin is determined by scoring adverse events, specifically coughing, and lung function measurement.

Nature and extent of the burden and risks associated with participation, benefit and group relatedness: All participants included in this study are patients recruited from the outpatient department of pulmonology. Before using the DPI they will receive instructions and their inspiratory flow will be tested. Before each test dose an infusion needle will be inserted

and after each test dose blood will be collected. To investigate safety, lung function tests will be performed and the occurrence of adverse events will be scored.

2. INTRODUCTION AND RATIONALE

Bronchiectasis

Bronchiectasis is a persistent and frequently progressive condition characterized by dilated and thick-walled bronchi. This pathology can result from many underlying conditions, including post-infectious conditions, like bacterial, mycobacterial (Lady Windermere Syndrome) and viral infections or aspergillus infections. Other causes are of congenital origin, like cystic fibrosis, primary ciliary dyskinesia, alpha-1-antitrypsin deficiency, or Mounier-Kuhns' and Marfan's syndromes; or immuno-deficiencies, both primary like Common Variable Immuno-Deficiency and secondary (e.g., due to hemato-oncological disease). Moreover, toxic inhalation, gastric or foreign body aspiration and rheumatic diseases, like rheumatoid arthritis, systemic lupus erythematosus or Sjögren's syndrome, can be a cause as well. Lastly, there is a large group of other conditions as cause of bronchiectasis. Examples of this group are allergic bronchopulmonary aspergillosis (ABPA), Yellow Nail Syndrome, and inflammatory bowel syndrome [1-4]

The main symptoms of bronchiectasis are cough and chronic sputum production. The sputum is described as mucoid, mucopurulent, thick, tenacious or viscous. Blood-streaked sputum or hemoptysis can result from erosive airway damage caused by an acute infection. The presence (colonization) of bacteria such as *P. aeruginosa* and *H. influenzae* can stimulate neutrophilic and inflammatory mediator responses in the airways [5](Angrill, Agusti et al. 2001). In different studies *H. influenza* was isolated in 29-42 percent and *P. aeruginosa* was isolated in 13-31 percent of the patients with steady state non-CF bronchiectasis [6,7]

Pseudomonas aeruginosa

P. aeruginosa is a gram-negative, motile, non-fermenting, obligate aerobic rod. It is commonly present in soil, water, sewage, plants and animals. Patients colonized with *P. aeruginosa* are a major reservoir and person-to-person contacts serve as an important mode of transmission, especially in hospitals. Respiratory infections due to transmission occur mostly in patients with impaired broncho-pulmonary function, like bronchiectasis, and systemic immune deficiencies.

The presence of *P. aeruginosa* in patients with bronchiectasis is associated with increased sputum production, more extensive bronchiectasis on HR-CT of the thorax, more hospitalizations and reduced quality of life [6,8-11]

Inhalation therapy in bronchiectasis

Nowadays treatment for patients having non-CF bronchiectasis who are colonized with *P. aeruginosa* consists of inhalation therapy with tobramycin or colistin in combination with oral macrolides. Tobramycin is in most cases administered by use of a nebulizer, for 28 days in 2 doses of 300 mg each. Thereafter, 28 days without therapy (or with colistin) follow, after which the cycles repeat. Occasionally, other pathogens like *Enterobacter* spp or mixed infections with similar clinical challenges and difficulties are encountered requiring a similar therapeutic approach. An alternative novel, commercial dry powder tobramycin device exists but has not been well characterized nor had it achieved broad market acceptance. No studies have been done in non CF-bronchiectasis, vide infra.

Tobramycin inhalation by wet nebulization

The currently broadly applied nebulisation technique for administration of antibiotics to patients with infectious lung diseases is ineffective, time consuming and therefore, burdensome to the patient. Furthermore, nebulisation brings the risk of auto-re-infection of the patient (contamination of nebulisation fluid and/or device). Another drawback is the low lung deposition, with several studies showing a mere 10% lung deposition of tobramycin using a nebulizer-compressor (de Boer, Hagedoorn et al. 2006). Nebulizers require space and time: carrying on during travel is problematic, and much time is required for nebulization, which is burdening and jeopardizing adherence with therapy. Dry powder inhalation of antibiotics with a more effective lung deposition can be an interesting alternative, as it can be simple, easy to carry-on, cheap and therefore disposable. This eliminates the need for regular disinfection while a cold chain for storage and the reconstitution of the drug is not needed [12].

Tobramycin inhalation by dry powder

Dry powder inhalation of tobramycin in higher dosages was first described by Geller et al. They studied tobramycin inhalation powder (TIP, delivered via the Podhaler device) in patients with CF, colonized with *P. aeruginosa*. Both safety and efficacy of a dose of 112 mg TIP compared to a dose of 300 mg nebulized tobramycin solution were studied with comparable pharmacokinetic parameters for both forms of inhalation [13]. A recent study, the EVOLVE trial, compared TIP to placebo in patients with CF colonized with *P. aeruginosa*. TIP reduced sputum *P. aeruginosa* density, respiratory related hospitalization and antipseudomonal antibiotic use [14]. Another study, the EAGER trial, compared 112 mg TIP to 300 mg nebulized tobramycin inhalation solution (TIS). Increases in FEV1% predicted from baseline to day 28 of cycle 3 were similar between groups. The mean reduction in sputum *P. aeruginosa* density (log10 CFU/g) on day 28 of cycle 3 was also comparable between groups. As expected, administration time was significantly less for TIP. Treatment

satisfaction was significantly higher for TIP on a scale of effectiveness, convenience, and global satisfaction. The rate of cough suspected to be study drug related was higher in TIP-treated patients (TIP: 25.3%; TIS: 4.3%) [15]. A disadvantage is the hygroscopicity of the TIP formulation. This makes TIP preservable for a maximum of 7 days. TIP is manufactured by Novartis and, is registered as TOBI® Podhaler®, and has been approved in the European Union by the Committee for Medicinal Products for Human Use (CHMP) in September 2010 for cystic fibrosis. It is now marketed and reimbursed in the Netherlands.

Novel dry powder inhaler (the Cyclops®)

Good performance of a dry powder inhaler (DPI) depends on the balance achieved between three types of forces, the interparticulate forces in the powder, the de-agglomeration forces during inhalation, and the deposition forces in the human lungs (see fig. 1).

The DPI used in this study was developed at the department of Pharmaceutical Technology and Biopharmacy of the University of Groningen. It was specifically designed to deliver high doses of dry powder drugs, for example antibiotics. Its dispersion principle is based on patented air classifier technology, which enables a reduction of the influence of the patient's breathing behavior on drug delivery (see fig. 2).

Background and justification over dry powder inhalation, especially with aminoglycosides, will be explained further in paragraph 5.6.3

Figure 1: Schematic overview of the balance between three types of forces to achieve a good performance of a dry powder inhaler.

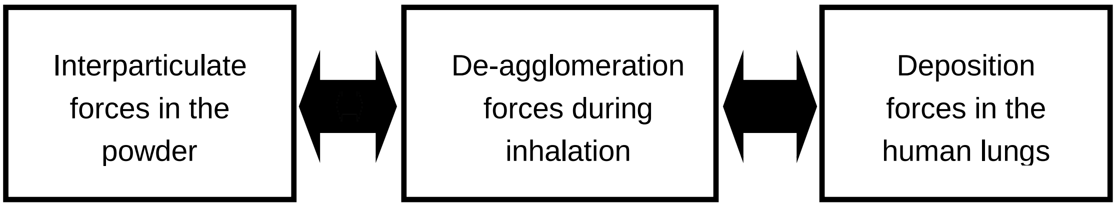

Figure 2. the Cyclops®

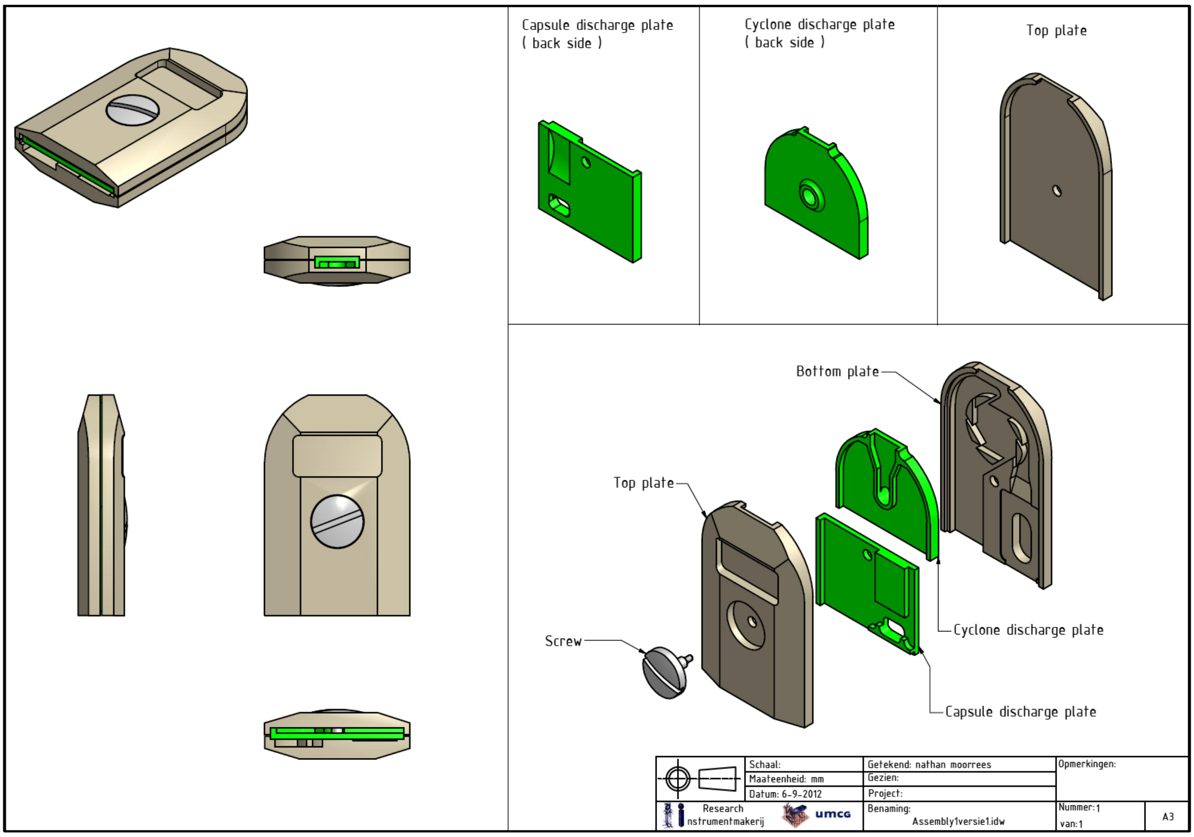

Tobramycin

The tobramycin formulation used consists of tobramycin free-base, whereas tobramycin sulphate is used in TOBI® nebulizer solution. Switching from one salt to another, or as in this case, to the unassociated form, is allowed when bioavailablity, pharmacokinetics and toxicological profile are equivalent. Both the free base and the sulphate are freely soluble in water. Therefore, we believe the free base will behave similarly when it enters the airways. Upon dissolution in the humid environment of the airways, tobramycin sulphate dissociates immediately, and tobramycin becomes available as free base. Presenting tobramycin free base directly or after immediate dissociation, presumably does not affect its pharmacokinetics. To assess pharmacokinetics and to rule out any chance on giving a dose that might induce unacceptable side effects, a safety and dose escalation regime is chosen for the pilot study, with sufficient time for complete washout prior to the administration of the next dose.

Current protocol

This study will investigate both the pharmacokinetic parameters and the local tolerability inhalation of dry powder tobramycin using the Cyclops® in patients with non-CF bronchiectasis with four different dosages. The first dose of 30 mg is chosen because this dose has the best dispersing properties with in vitro experiments. This last dose (240 mg) is generally considered safe, and it is still lower than the dose normally given as first dose intravenously in patients with sepsis of unknown origin.

Tobramycin by nebulization for non-CF bronchiectasis is routinely administered at a dose of 300 mg twice a day. Until now, as far as we know, no studies have investigated inhalation of tobramycin by nebulisation or as dry powder in patients with non-CF bronchiectasis colonized with *P. aeruginosa* (the studies with the Novartis compound mentioned earlier were in CF-bronchiectasis, a different disease at generally much younger age). In patients with CF bronchiectasis tobramycin inhalation powder showed similar results with a dose of 112 mg compared to a dose of 300 mg nebulized tobramycin solution[13].

However, as we are most interested in clinical and microbiological response, an efficacy and safety study will be planned after the current porposal. That study will compare TOBI® inhalation solution (the current routine treatment) and dry powder inhalation of tobramycin via the Cyclops® in different treatment strategies. The treatment regimen of DP tobramycin using the Cyclops® will be chosen based on the PK parameters of this study

3. OBJECTIVES

As we described previously, within the population of patients with bronchiectasis, colonization with *P. aeruginosa* is associated with increased sputum production, more extensive bronchiectasis on HR-CT of the thorax, more hospitalizations and reduced quality of life. In current guidelines non-CF bronchiectasis patients colonized with *P. aeruginosa* need to be treated with antibiotics, for example nebulized tobramycin. Occasionally, other organisms as well as mixed infections result in similar problems, and these patients may also benefit from inhaled tobramycin.
The objective of this study is to document pharmacokinetics and local tolerability of dry powder tobramycin inhaled by patients with non-CF bronchiectasis by administering 4 different doses of tobramycin: 30, 60, 120 and 240 mg.

Primary objectives
To establish both pharmacokinetics and local tolerability of DP tobramycin using the Cyclops, in the target population.

4. STUDY DESIGN

The study will be a single centre, single ascending, single dose study of inhaled dry powder tobramycin as a first step to investigate the pharmacokinetics of four doses with no more local intolerability than when tobramycin is administered by nebulisation. The study includes 8 patients with non-CF bronchiectasis who meet the in- and exclusion criteria. These 8 patients will be recruited from the outpatient department of pulmonology.

See tables 1 and 2 for interventions and a timetable

Table 1 Study procedures

	Procedure	Extra for study	Invasive	For study objective
Medication	Study medication	X	N	primary
Measurements	Inhalation instruction	X	N	primary
	Inhalation flow measurement	X	N	primary
	Serum levels	X	Y	primary
	Serum creatinin	X	Y	primary
	Lung function	X	N	primary
	X-ray of the chest, if not performed the last year before screening	X	N	primary
Medical check	Routine check up	X	N	

Table 2. Timetable of the study

Visit	Time table	Time needed	Procedure
1	Day 1	15 minutes	Explanation study design and invitation to participate in the study
2	Day 15	30 minutes	Informed consent<br>Routine medical check, including spirometry and if necessary a X-ray of the chest
3	Day	13 hours	Inhalation instruction<br>Medication inhalation, 1st dose = 30 mg<br>Inhalation flow measurement<br>Collecting inhalation device<br>Measuring amount of tobramycin remaining in the device<br>Serum samples collection – from indwelling venous catheter<br>Spirometry<br>Scoring of adverse effects
4	Day 7 after visit 3	13 hours	Inhalation instruction<br>Medication inhalation, 2nd dose = 60 mg<br>Inhalation flow measurement<br>Collecting inhalation device<br>Measuring amount of tobramycin remaining in the device<br>Serum samples collection – from indwelling venous catheter<br>Spirometry<br>Scoring of adverse effects
5	Day 7 after visit 4	13 hours	Inhalation instruction<br>Medication inhalation, 3rd dose = 120 mg<br>Inhalation flow measurement<br>Collecting inhalation device<br>Measuring amount of tobramycin remaining in the device<br>Serum samples collection – from indwelling venous catheter<br>Spirometry<br>Scoring of adverse effects
6	Day 7 after visit 5	13 hours	Inhalation instruction<br>Medication inhalation, 4th dose = 240 mg<br>Inhalation flow measurement<br>Collecting inhalation device<br>Measuring amount of tobramycin remaining in the device<br>Serum samples collection – from indwelling venous catheter<br>Spirometry<br>Scoring of adverse effects

5. STUDY POPULATION

5.1 Population (base)

All participants will be patients with non-CF bronchiectasis and will be recruited via the outdoor department of the department of pulmonary diseases of the UMCG. Due to the low number of study participants needed to perform this study we expect to reach the target number of patients needed for this study within several months.

5.2 Inclusion criteria

- Age 18 years or older
- Obtained informed consent
- Patients having bronchiectasis (confirmed with HR-CT of the chest)

5.3 Exclusion criteria

- Pregnant or breast feeding
- Subjects with known or suspected renal, auditory, vestibular or neuromuscular dysfunction, or with severe, active haemoptysis,
- History of adverse events on previous tobramycin or other aminoglycoside use
- No concurrent use of cyclosporin, cisplatin, amfotericin B, cephalosporins, polymyxins, vancomycin and NSAIDs.

5.4 Sample size calculation

We assume to need eight participants for this study. A specific calculation of the number of subjects needed is difficult, but low numbers have provided good results in other dry powder inhalation studies of antibiotics: A single dose study with dry powder inhalation of colistin for cystic fibrosis (CF) patients by LeBrun et al was performed with 5 patients. Another single dose study with dry powder colistin inhalation by Westerman et al was performed with 10 CF patients. Geller et al performed a single dose with a dose escalation study of dry powder tobramycin inhalation with 12 CF patients in each dose escalation arm. Each arm inhaled a fixed dose. Newhouse et al did a 5 period study with technetium labeled dry powder tobramycin with 14 healthy volunteers. First 3 periods were single dose. The last period was a 6-fold dose. Pilcer et al performed a single dose study with dry powder tobramycin inhalation in 9 CF patients. Therefore, we expect the 8 subjects in our single ascending dose study to be sufficient[12,13,16-18].

6. INVESTIGATIONAL PRODUCT

6.1 Name and description of investigational product(s)

Dry powder (DP) tobramycin consists of pure spray dried tobramycin free base. It is a white to off-white, hygroscopic powder with a particle size distribution appropriate for pulmonary administration. One dose of 30 mg tobramycin is sealed in an aluminium blister. Coarse crystalline α-lactose monohydrate particles are added to the blister (not blended with the tobramycin) to break up any agglomerate that forms during storage.

6.2 Summary of findings from non-clinical studies

Preclinical data show that target organs for tobramycin toxicity are the kidneys and the vestibular/cochlear functions. This toxicity was shown at systemic tobramycin levels that were higher than can be achieved by inhalation of the recommended clinical dose given by nebulisation (Summary of Product Characteristics (SPC) TOBI®, SPC Bramitob®). Administration of inhaled tobramycin during up to 28 consecutive days might give rise to modest and unspecific signs of irritation of the respiratory tract, that were fully reversible on therapy discontinuation (SPC Bramitob®).

Subcutaneous administration at doses of 100 mg/kg/day in rats and 20 mg/kg/day in rabbits were not teratogenic during organogenesis. Higher parenteral doses induced maternal toxicity and abortion in rabbits and were not relevant to be assessed on teratogenicity (SPC TOBI®, SPC Bramitob®).

No genotoxicity was shown for tobramycin (SPC Bramitob®).

6.3 Summary of findings from clinical studies

Several studies in the CF population with inhaled tobramycin were done in the late eighties and early nineties of the last century with promising results [19]. However not all therapies that are effective in CF are effective in non-CF bronchiectasis [20].

Later, studies with tobramycin solution for inhalation were performed in patients with non-CF bronchiectasis. Barker et al investigated 74 patients divided in 2 groups. 37 patients received 300 mg tobramycin solution via a nebulizer twice daily for 28 days, the others received placebo. The patients treated with tobramycin had a significant reduction in sputum *P. aeruginosa* density at all time points of the study. The placebo group had a negligible change in sputum density at all time points. Unfortunately, the incidence of dyspnoea, chest pain and wheezing was significantly greater in the tobramycin group [1].

Orriols et al studied 17 patients with non-CF bronchiectasis colonised with *P. aeruginosa*, divided in 2 groups. Group A received both tobramycin and ceftazidim via nebulisation inhalation and group B was treated symptomatically. One patient in group A withdrew due

to bronchospasm despite intense bronchodilator treatment. One patient in group B died due to respiratory failure after 300 days of follow up. No differences in lung function or arterial blood gas analysis were seen between the two groups. There was only one hospital admission in group A and 7 patients who had to be admitted in group B. In both groups *P. aeruginosa* was still present. They did not study sputum density [21].
Couch et al studied 74 patients with non-CF bronchiectasis colonized with *P. aeruginosa*. Patients were evenly divided in two groups, one group is receiving tobramycin inhalation therapy and the other group receives placebo. The treatment group received tobramycin for 4 weeks 300 mg twice daily. This group had a significant reduction in sputum *P. aeruginosa* density while the placebo group showed no change. The incidence of dyspnoea, wheezing and chest tightness was again significantly greater in the tobramycin group [22].

6.4 Summary of known and potential risks and benefits

6.4.1. Risks (SPC TOBI®, SPC Bramitob®)

Tobramycin, as all aminoglycosides, should be used cautiously in patients with known or suspected renal, auditory, vestibular or neuromuscular dysfunction, or with severe, active haemoptysis. Serum concentrations of tobramycin should be monitored through venipuncture. Parenteral aminoglycosides have been associated with hypersensitivity, ototoxicity and nephrotoxicity. No evidence of nephrotoxicity or of ototoxicty was shown during clinical trials with inhaled tobramycin.
Patients with neuromuscular disorders or other conditions characterized by myasthenia, should receive therapy with inhaled tobramycin with great caution because tobramycin might aggravate muscle weakness.
Bronchospasm has been reported with nebulised tobramycin. Furthermore, inhalation of medicinal products might evoke a cough reflex, that has been shown to occur in treatment with both nebulised tobramycin as with Tobramycin Inhalation Powder [15]. Cough might induce further haemorrhage in patients with active haemoptysis.
In controlled clinical trials with TOBI®, dysphonia and tinnitus were the only unwanted effects reported in significantly more patients treated with TOBI®. In controlled clinical trials with Bramitob, most common adverse events were those concerning the respiratory tract (cough, dyspnoea, increased expectoration, FEV1 reduction). Furthermore, nausea is reported as common adverse event (occurrence >1/100).

6.4.2. Benefits

Intermittent use of nebulized tobramycin is indicated for the long-term management of chronic pulmonary infection due to *P. aeruginosa* in CF patients aged 6 years and older (SPC TOBI®). Several studies (see paragraph 6.3) showed that treatment with inhaled tobramycin might also be beneficial to patients with non-CF bronchiectasis whom are colonized by *P. aeruginosa*. Long-term intermittent administration appears to be safe and lessen disease severity.

Benefits of administering tobramycin by use of the DPI instead of by nebulisation are a less time consuming and easier drug administration that is less of a burden on the patient's (social) life and no pollution of the environment. Furthermore, because it is a single-use device there is no need for hygiene control or maintenance of the equipment and no risk of device contamination and patient (re)infection.

6.5 Description and justification of route of administration and dosage

The way of administration will be as dry powder using a DPI. Further information about the technical aspects of the DPI see section *The dry powder inhaler* in the Introduction and rationale. This device can generate high volumes of dry powder, needed for a substance and with an aim like tobramycin.

6.6 Dosages, dosage modifications and method of administration

In a study with CF patients 112 mg of dry powder tobramycin using the Podhaler was comparable with 300 mg of tobramycin via nebulisation, which is the recommended clinical dose in this population [13-15]. However for patients with non-CF bronchiectasis no optimal dosage has been studied.
Based on the in vitro data analysis, we expect that a lower dose of DP tobramycin using the Cyclops® will result in similar deposition characteristics with similar pharmacokinetic parameters compared to DP tobramycin using the Podhaler. Since, the Cyclops® has a higher inhaler resistance than the Podhaler®, the airflow through the Cyclops® (at the same pressure drop) will be lower and this will result in a more efficient lung deposition and thus, we expect that a lower tobramycin dose will result in the same plasma levels as the Podhaler® dose of 112 mg. To test this hypothesis it's not necessary to incorporate a DP Tobramycin dose of 112 mg in the Cyclops®, because the inhalers and the formulations are very different. Furthermore, the Cyclops® is optimised to disperse 30 mg of DP Tobramycin (blisters contain 30 mg), therefore the doses chosen are 30, 60, 120 and 240 mg. Two dosages are below the Podhaler dosage, one is more or less comparable with the current dosage of the Podhaler and the last dose is higher than the

dosage of the Podhaler to study the local tolerability of the Cyclops® in case of an overdose. This last dose (240 mg) is generally considered safe, and it is still lower than the dose given normally as first dose intravenously in patients with sepsis of unknown origin.

6.6.1. Dosages:

We will do an ascending dose response study with 4 different doses (30, 60, 120 and 240 mg). The doses will be fixed rising to react to possible adverse events and a possible end of the study for the participant.

6.6.2. Method of administration:

Tobramycin will be administered by inhalation using a DPI, called the Cyclops®. The DPI has been developed at the department of Pharmaceutical Technology and Biopharmacy of the University of Groningen.

6.6.3. Justification of the new dry powder inhalation device, the Cylops®

Aminoglycosides (like tobramycin) are very hygroscopic. The delivery with re-usable capsule inhalers can cause particles to retain within the inhaler, which can absorb moisture from the air and stick firmly to the inhaler walls and this can influence following inhalations.

The Cyclops® is designed as a disposable device with a high inhaler resistance to reduce the flow rate, and thus, the speed of entry of the tobramycin particles. This will significantly reduce the mouth and throat deposition, and increase the dose fraction available for deposition in the lungs.

To reach the highest deposition in the peripheral airways (94% of total lung volume is in the generations 17 to 23), the total dose has to enter the airways within the first 0.5 to 1 L of air inhaled. When the emission time is longer than two seconds at a flow rate of 30 L/min, more than 1 L of air has to be inhaled to take the entire dose. The emission time from the Cyclops® is less than 2 seconds. Table 3 shows that the dispersion efficiency of the Cyclops®.

The formulation used in the Cyclops® consists of 100 per cent tobramycin. Because this inhaler was specifically designed for tobramycin, no excipients are needed to disperse the formulation. Moreover, because no excipients are needed, a single blister can contain a much higher fraction of the total drug dose.

In conclusion, the specific design of the Cyclops® with the high inhaler resistance (that prevents high mouth and throat deposition), and the patented air classifier technology, will result in an efficient dispersion, and thus probably in a more efficient therapy.

Table 3: Dispersion efficiency of the Cyclops® and TOBI® Podhaler® at a flow rate of 30 L/min.

	Cyclops® tobramycin<br>50 mg dose (=50 mg tobra)<br>30 L/min.	TOBI® Podhaler®<br>45.5 mg dose (=28 mg tobra)<br>30 L/min.
X_{10} (µm)	1.75	1.94
X_{50} (µm)	3.26	3.95
X_{90} (µm)	6.38	9.71

6.7 Preparation and labelling of Investigational Medicinal Product

Preparation:

A solution of 50 mg/mL tobramycin in water for injections was spray dried with a Büchi Mini B-290 spray dryer. The spray dried product was transferred to a freeze-dryer (Christ, Epsilon 2-4) to remove residual water. Tobramycin is very hygroscopic and water can cause the formation of agglomerates, which can negatively affect powder dispersion properties.

Filling:

Aluminum blisters were filled with 30 mg of tobramycin and 10 mg of α-lactose monohydrate sweeper crystals (Ph.Eur. quality; DMV Fonterra Excipients, Goch, Germany) to obtain the final DP Tobramycin product.

Sealing:

The blisters are sealed thermally with standard sealing equipment. The seal is formed between a PVC coating on the aluminum blister and a heat lacquer (suitable for PVC) on the aluminum foil strip by heating to around 200 °C for a few seconds.

Labelling:

Labels will contain the following information:

a) name, address and telephone number of the investigator;
b) pharmaceutical dosage form, route of administration, quantity of dosage units;
c) the batch number;
d) a trial reference code;

e) “for clinical trial use only” or similar wording;
f) the storage conditions;
g) expiry date.

6.8 Drug accountability

Drug accountability will be performed according GCP guidelines and performed by the investigator and the Hospital Pharmacy.

7. METHODS

7.1 Study parameters/endpoints

7.1.1 Main study parameter/endpoint

The following pharmacokinetic parameters will be calculated:

- Actual dose (dose minus remainder in inhaler after inhalation)
- AUC_{0-12} (area under the curve from 0 -12 h)
- C_{max} (maximum plasma concentration)
- T_{max} (time to maximum plasma concentration)
- Ka absorption rate constant
- $T_{1/2\,el}$ terminal elimination half-life
- CL/F clearance following pulmonary administration (F= bioavailability)

Local tolerability of the inhalation of dry powder tobramycin. Both points need to have a positive result.

- Drop of FEV1 of >10% (lung function measurement)
- Adverse events

7.2 Randomisation, blinding and treatment allocation

Not applicable

7.3 Study procedures

7.3.1 Pharmacokinetic analysis

Pharmacokinetic analysis is studied using software suitable for population pharmacokinetic modeling (i.e. MW/Pharm NonMem)[23].

7.3.2 Actual inhaled dose

After inhalation the inhaler will be tested for the amount of drug remaining in the device. The device will be rinsed with water and the remaining tobramycin will be determined using a modification of the 2,4,6-Trinitrobenzene Sulfonic Acid (TNBSA) assay. The actual inhaled dose of tobramycin will be calculated by subtracting the amount of drug remaining in the device after inhalation from the total dose weighed into the device.

7.3.3 Blood sampling and analysis:

Venous blood samples will be collected after inhalation of tobramycin from an infusion needle. Serum samples will be collected directly before and after 15 minutes, 30 minutes, 45 minutes, 1 h, 1¼ h, 1½ h, 1¾ h, 2 h, 4 h, 8 h and 12 h after inhalation. These samples

will be centrifuged immediately and the serum will be stored at -20°C during the sampling day (UMCG, Haren). After shipment to the UMCG (Groningen) samples will be stored at -80C until analysis.

7.3.4 Gastro-intestinal absorption of tobramycin

From the literature it is expected that the gastro-intestinal absorption of tobramycin is far less than 1%. Therefore, we will not test the gastro-intestinal absorption in this group of subjects.

7.3.5 Serum creatinin analysis

Serum creatinin will be determined in the routine UMCG central lab.

7.3.6 Creatinin clearance

Will be calculated using the formula of Cockroft-Gault

7.3.7 Adverse effects

Spirometry will be used to test for adverse broncoconstrictive reactions due to the inhalation, immediately before, 15, 30 and 90 minutes after inhalation. It will be performed by standardized procedure according to the ERS guidelines and reference values. A 10% or more decrease in FEV1 (in mL) will be considered clinically relevant.
Other adverse effects will be asked by the investigator for spontaneous reporting. Special attention will be for cough and dyspnea.

7.3.8 X-ray of the chest

Before the start of the study a X-ray of the chest will be made of the patients who haven’t had one the last year before screening for this study. This is both to find no existing abnormalities before start of the study and to have a reference in case an adverse event has happened.

7.3.9 Inhalation flow measurement

Prior to inhalation the patients will receive inhalation instructions and the opportunity to practice the inhalation manoeuvre. This will be done by using an empty inhaler connected to an electronic inspiratory flow measurement device (University of Groningen). With this device inhalation flow curves will be recorded and as soon as a series of consistent maneuvers is obtained, the Cyclops® containing tobramycin is given to the patient for administration of the drug. During the inhalation of tobramycin the inspiratory flow will be measured as well.

7.4 Withdrawal of individual subjects

Subjects can leave the study at any time for any reason if they wish to do so without any consequences. The investigator can decide to withdraw a subject from the study for urgent medical reasons.

7.4.1 Specific criteria for withdrawal

Special criteria for withdrawal include an allergic or anaphylactic reaction to tobramycin, and drop in FEV_1 > 10%, cough or dyspnoea in direct relation to the inhalation given the rising dose administration.

7.5 Replacement of individual subjects after withdrawal

If a subject withdraws him or herself, he or she will be replaced in order to reach the number of subjects needed for this study.

7.6 Follow-up of subjects withdrawn from treatment

Follow-up will be done by their chest physician or their general practitioner

7.7 Premature termination of the study

The criteria for terminating the study prematurely are a high percentage of adverse events like allergic reactions, and a high number of withdrawals for whatever reason.

8. SAFETY REPORTING

8.1 Section 10 WMO event

In accordance to section 10, subsection 1, of the WMO, the investigator will inform the subjects and the reviewing accredited METC if anything occurs, on the basis of which it appears that the disadvantages of participation may be significantly greater than was foreseen in the research proposal. The study will be suspended pending further review by the accredited METC, except insofar as suspension would jeopardise the subjects' health. The investigator will take care that all subjects are kept informed.

8.2 AEs, SAEs and SUSARs

8.2.1 Adverse events (AEs)

Adverse events are defined as any undesirable experience occurring to a subject during the study, whether or not considered related to [the investigational product / the experimental interventiont]. All adverse events reported spontaneously by the subject or observed by the investigator or his staff will be recorded.

8.2.2 Serious adverse events (SAEs)

A serious adverse event is any untoward medical occurrence or effect that at any dose:

- results in death;
- is life threatening (at the time of the event);
- requires hospitalisation or prolongation of existing inpatients' hospitalisation;
- results in persistent or significant disability or incapacity;
- is a congenital anomaly or birth defect;
- Any other important medical event that may not result in death, be life threatening, or require hospitalization, may be considered a serious adverse experience when, based upon appropriate medical judgement, the event may jeopardize the subject or may require an intervention to prevent one of the outcomes listed above.

The sponsor will report the SAEs through the web portal *ToetsingOnline* to the accredited METC that approved the protocol, within 15 days after the sponsor has first knowledge of the serious adverse reactions.

SAEs that result in death or are life threatening should be reported expedited. The expedited reporting will occur not later than 7 days after the responsible investigator

has first knowledge of the adverse reaction. This is for a preliminary report with another 8 days for completion of the report.

8.2.3 Suspected unexpected serious adverse reactions (SUSARs)

Adverse reactions are all untoward and unintended responses to an investigational product related to any dose administered.

Unexpected adverse reactions are SUSARs if the following three conditions are met:

1. the event must be serious (see chapter 9.2.2);
2. there must be a certain degree of probability that the event is a harmful and an undesirable reaction to the medicinal product under investigation, regardless of the administered dose;
3. the adverse reaction must be unexpected, that is to say, the nature and severity of the adverse reaction are not in agreement with the product information as recorded in:
   - Summary of Product Characteristics (SPC) for an authorised medicinal product;
   - Investigator's Brochure for an unauthorised medicinal product.

The sponsor will report expedited the following SUSARs through the web portal *ToetsingOnline* to the METC:

- SUSARs that have arisen in the clinical trial that was assessed by the METC;
- SUSARs that have arisen in other clinical trials of the same sponsor and with the same medicinal product, and that could have consequences for the safety of the subjects involved in the clinical trial that was assessed by the METC.

The remaining SUSARs are recorded in an overview list (line-listing) that will be submitted once every half year to the METC. This line-listing provides an overview of all SUSARs from the study medicine, accompanied by a brief report highlighting the main points of concern.
The expedited reporting of SUSARs through the web portal ToetsingOnline is sufficient as notification to the competent authority.

The sponsor will report expedited all SUSARs to the competent authorities in other Member States, according to the requirements of the Member States.

The expedited reporting will occur not later than 15 days after the sponsor has first knowledge of the adverse reactions. For fatal or life threatening cases the term will be maximal 7 days for a preliminary report with another 8 days for completion of the report.

8.3 Annual safety report

As we expect to end this study within one year, a safety report will be submitted after the study is ended to the accredited METC, competent authority, Medicine Evaluation Board and competent authorities of the concerned Member States.
This safety report consists of:

- a list of all suspected (unexpected or expected) serious adverse reactions, along with an aggregated summary table of all reported serious adverse reactions, ordered by organ system, per study;
- a report concerning the safety of the subjects, consisting of a complete safety analysis and an evaluation of the balance between the efficacy and the harmfulness of the medicine under investigation.

8.4 Follow-up of adverse events

All AEs will be followed until they have abated, or until a stable situation has been reached. Depending on the event, follow up may require additional tests or medical procedures as indicated, and/or referral to the general physician or a medical specialist. SAEs need to be reported till end of study within the Netherlands, as defined in the protocol

8.5 Data Safety Monitoring Board (DSMB) / Safety Committee

Will not be established for this very short time, 8 subject study of a new device but with a well known drug.

9. STATISTICAL ANALYSIS

9.1 Descriptive statistics

Descriptive statistics will be used for calculation (geometric) mean and (interquartile) range of observed age, weight, length, dose, clinical parameters, (unbound) $AUC_{0\text{-}24h}$, and Cmax/MIC ratio, (unbound) $AUC_{0\text{-}24h}$/MIC, T_{max}, C_{max}, $T_{1/2}$ will be calculated with a 95% confidence interval. The other end-point will be proportions of subjects reporting adverse effects on all treatment days and / or percentage of drop of FEV1 of more than 10%.

9.2 Inferential statistics

Univariate analysis will be used to test if co-variables, including sex, ethnicity, co-morbidity, medical history, dose and duration of (TB) and co medication have an effect on PK parameters like T_{max}, C_{max} and the (unbound) $AUC_{0\text{-}24h}$ of tobramycin. Differences in clinical parameters between baseline and end of study will be calculated. When not normally distributed, non-parametric tests were used, i.e. Mann-Whitney *U* test and Wilcoxon rank sum test for ordinal data and Chi square tests will be used for nominal data. Correlation coefficients (Spearman's or Pearson's depending on distribution) will be used to test

9.3 Multivariate analysis

Multivariate analyses will be used to test relations between $AUC_{0\text{-}24h}$ and the tobramycin dose corrected for bodyweight and potential other confounders.

10. ETHICAL CONSIDERATIONS

10.1 Regulation statement

This study will be conducted according to the principles of the Declaration of Helsinki (version 9, October 2008, www.wma.net) and in accordance with the Medical Research Involving Human Subjects Act (WMO).

10.2 Recruitment and consent

Participants will be recruited from the outpatient clinic of pulmonology. First, patients will be contacted by their physician. If patients are willing to participate, they are referred by their treating physician to the investigator. Their treating physician gives the intended participant the subject information brochure of this study. On day 1 of the contact between patient and investigator (see flow diagram page 15), in- and exclusion criteria will be checked, and the –study participants will be informed about the nature and goals of the study, as well as the advantages and disadvantages of study participation. Questions will be answered and sufficient time (2 weeks) will be offered. If participants so wish they will be offered the opportunity to see an independent physician. Both the participant and the investigator will sign the informed consent if the patient is willing to participate.
For the informed consent and the subject information letter see attachments.

10.3 Benefits and risks assessment, group relatedness

No direct benefits to participants are expected. The risks are deemed relatively minor; there could be transient cough or dyspnea reactions immediately following inhalation.

10.4 Compensation for injury

The UMCG has a liability insurance that is in accordance with article 7, subsection 6 of the WMO.

The UMCG (also) has an insurance which is in accordance with the legal requirements in the Netherlands (Article 7 WMO and the Measure regarding Compulsory Insurance for Clinical Research in Humans of 23th June 2003). This insurance provides cover for damage to research subjects through injury or death caused by the study.

1. € 450.000,-- (i.e. four hundred and fifty thousand Euro) for death or injury for each subject who participates in the Research;
2. € 3.500.000,-- (i.e. three million five hundred thousand Euro) for death or injury for all subjects who participate in the Research;

3. € 5.000.000,-- (i.e. five million Euro) for the total damage incurred by the organisation for all damage disclosed by scientific research for the Sponsor as ‘verrichter’ in the meaning of said Act in each year of insurance coverage.

The insurance applies to the damage that becomes apparent during the study or within 4 years after the end of the study.

10.5 Incentives

Subjects whom participating the study after inclusion will receive 100 euro in gift certificates per visit. This apllies only to the visits where the participant is inhaling the tobramycin. This amounts to the minimum wage of 8,38 Euro per hour. They will also receive meals during their visit in the hospital and their travel expenses. For the first visit, checking the in- and exclusion criteria and explanation of the study, the participant will receive 10 euro in gift certificates. For the second visit, signing the informed consent and undergo a medical check and perform a spirometry, they will receive 15 euro in gift certificates.

11. ADMINISTRATIVE ASPECTS, MONITORING AND PUBLICATION

11.1 Handling and storage of data and documents

Handling of the personal data in this observational study will comply with the Dutch Personal Data Protection Act. Data will be handled confidentially and anonymously. To trace the data of individual patients, the study will use a subject identification code list that is linked to the date of the participating patients. The code will not be based on the patient initials and birth date, but on number of enrolment into the study. At the end of the study, all data will be kept for of 20 years

11.2 Monitoring and Quality Assurance

Not applicable

11.3 Amendments

A ‘substantial amendment’ is defined as an amendment to the terms of the METC application, or to the protocol or any other supporting documentation, that is likely to affect to a significant degree:

- the safety or physical or mental integrity of the subjects of the trial;
- the scientific value of the trial;
- the conduct or management of the trial; or
- the quality or safety of any intervention used in the trial.

All substantial amendments will be notified to the METC and to the competent authority.

Non-substantial amendments will not be notified to the accredited METC and the competent authority, but will be recorded and filed by the sponsor.

11.4 Annual progress report

As we expect to end this study within one year, the sponsor/investigator will submit a final report of the trial to the accredited METC after terminating the study. Information will be provided on the date of inclusion of the first subject, numbers of subjects included and numbers of subjects that have completed the trial, serious adverse events/ serious adverse reactions, other problems, and amendments.

11.5 End of study report

The sponsor will notify the accredited METC and the competent authority of the end of the study within a period of 90 days. The end of the study is defined as the last patient's last visit.

In case the study is ended prematurely, the sponsor will notify the accredited METC and the competent authority within 15 days, including the reasons for the premature termination.

Within one year after the end of the study, the investigator/sponsor will submit a final study report with the results of the study, including any publications/abstracts of the study, to the accredited METC and the Competent Authority.

11.6 Public disclosure and publication policy

All investigators and sponsor are part of the UMCG and/or Rijksuniversiteit Groningen. They have agreed between them that results will be submitted for publication with joint authorship. The trial will be added in a public registry at www.Clinicaltrials.gov.

12. STRUCTURED RISK ANALYSIS

12.1 Potential issues of concern

a. Level of knowledge about mechanism of action

Tobramycin is an antibiotic drug from the pharmacotherapeutic group of aminoglycoside antibacterials which are useful in infections caused by aerobic Gram-negative bacteria, including *P. aeruginosa*. It is derived from the aminoglycoside antibiotic complex nebramycin produced by a strain of *Streptomyces tenebrarius*.

The exact mechanism of action of aminoglycosides is not known. Binding to the bacterial 30S (also 50S) ribosomal subunit, inhibiting the translocation of the peptidyl-tRNA from the A-site to the P-site, as well as misreading of mRNA have been reported, which leaves the bacterium unable to synthesize proteins that are vital to its growth [24].

b. Previous exposure of human beings with the test product(s) and/or products with a similar biological mechanism

No clinical studies have been performed with DP tobramycin yet. However, various studies have described the pulmonary administration of tobramycin, both administered by nebulisation as by dry powder inhalation. The earliest studies were performed in the late 1980s and early 1990s. TOBI® nebulizer solution, Bramitob® nebulizer solution and TOBI® Podhaler® inhalation powder are all registered medicinal products indicated for the treatment of colonization and chronic infections of the lung by sensitive *P. aeruginosa* strains in CF patients aged 6 years and older. In patients with non-CF bronchiectasis *P. aeruginosa* colonization is associated with clinical deterioration and these patients are treated with inhaled tobramycin as well. In practice, the same posology is applied in non-CF bronchiectasis patients as in CF patients.

Several studies in the CF population with inhaled tobramycin were done in the late 1980s and early 1990s with promising results [19,25-27]. However not all therapies that are effective in CF are effective in non-CF bronchiectasis [20].

Later, studies with tobramycin solution for inhalation were performed in patients with non-CF bronchiectasis. Barker et al. investigated 74 patients divided in two groups. 37 patients received 300 mg tobramycin solution via a nebulizer twice daily for 28 days, the others received placebo. The patients treated with tobramycin had a significant reduction in sputum *P. aeruginosa* density at all time points of the study. The placebo group had a negligible change in sputum density at all time points. Unfortunately, the incidence of dyspnoea, chest pain and wheezing was significantly greater in the tobramycin group[1].

Orriols et al. studied 17 patients with non-CF bronchiectasis colonized with *P. aeruginosa*, divided in two groups. Group A received both tobramycin and ceftazidim via nebulisation inhalation and group B was treated symptomatically. One patient of group A withdrew due to bronchospasm despite intense bronchodilator treatment. One patient of group B died due to respiratory failure after 300 days of follow up. No differences in lung function or arterial blood gas analysis were seen between the two groups. There was only one hospital admission in group A and seven patients of group B who had to be admitted. In both groups *P. aeruginosa* was still present. They did not study sputum density [21].
Couch et al. studied 74 patients with non-CF bronchiectasis colonized with *P. aeruginosa*. Patients were evenly divided in two groups, one group received tobramycin inhalation therapy and the other group received placebo. The treatment group received tobramycin for four weeks 300 mg twice daily. This group had a significant reduction in sputum *P. aeruginosa* density while the placebo group showed no change. The incidence of dyspnoea, wheezing and chest tightness was again significantly greater in the tobramycin group [22].

c. Can the primary or secondary mechanism be induced in animals and/or in *ex-vivo* human cell material?

No, the main study parameters/endpoints are AUC_{0-12} (area under the curve from 0 -12 h), C_{max} (maximum plasma concentration), T_{max} (time to maximum plasma concentration), Ka (absorption rate constant), $T_{1/2\ el}$ (terminal elimination half-life), CL/F (clearance following pulmonary administration (F= bioavailability)). Furtermore, local tolerability of DP tobramycin will be determined by lung function measurement and questioning for adverse events. These data can only be obtained by a study in patients.

d. Selectivity of the mechanism to target tissue in animals and/or human beings

Preclinical safety studies performed with tobramycin are safety pharmacology, repeated dose toxicity, genotoxicity, and toxicity to reproduction studies. Renal toxicity and ototoxicity are the main hazards of tobramycin for humans. These toxicities are mainly seen when high systemic levels of tobramycin are reached and it is unlikely that these levels will be reached after pulmonary administration at the doses administered in this study. No increase in carcinogenicity or genotoxicity was found

e. Analysis of potential effect

Ramsey et al. investigated 600 mg tobramycin in total divided in t.i.d. This showed clinical effectiveness without serious adverse events. After this study the dose administered was compared with this dose [19].

Barker et al. investigated 74 patients divided in two groups. 37 patients received 300 mg tobramycin solution via a nebulizer twice daily for 28 days, the others received placebo. The patients treated with tobramycin had a significant reduction in sputum *P. aeruginosa* density at all time points of the study. The placebo group had a negligible change in sputum density at all time points. Unfortunately, the incidence of dyspnoea, chest pain and wheezing was significantly greater in the tobramycin group [1].

f. Pharmacokinetic considerations

DP tobramycin has not been evaluated in clinical studies yet. The clinical pharmacokinetics and safety profile of nebulised tobramycin and parenterally administered tobramycin is knowledge which is available in the public domain. In addition, the TOBI® Podhaler® with a dry powder tobramycin formulation is already market approved. The pharmacokinetics and local tolerability of DP tobramycin are the objectives of this presented clinical study.

g. Study population

Patients with non-CF bronchiectasis, with recurrent bacterial exacerbations (i.e., ≥2 in previous year) – with or without *Pseudomonas aeruginosa* isolated from sputum.

h. Interaction with other products

The risk for nephrotoxicity is particularly increased by concurrent therapy with cyclosporin, cisplatin, and amfotericin B. This risk may also be increased when tobramycin is combined with certain cephalosporins, polymyxins, vancomycin and NSAIDs. Use of these products should be ceased if possible (NSAIDs), otherwise the patient cannot participate in the study.

i. Predictability of effect

The pharmacokinetic data gained can be compared with data from literature. Tobramycin has been used for decades to treat bacterial infections.
The efficacy of DP tobramycin will not be investigated during this study, only pharmacokinetic data will be gathered to be able to estimate the systemic toxicity of DP tobramycin.

j. Can effects be managed?

The study is designed in such a way that the risks are as low as possible (dose response). During this study DP Tobramycin will only be administered in a hospital setting, so in case of an emergency adequate action can be taken (e.g. salbutamol for bronchoconstriction).

12.2 Synthesis

The study design is chosen as such to minimize the chance on the occurrence of an adverse event. The starting dose is much lower than the dose given with TOBI Podhaler®, because we expect that the fraction of the dose deposited in the lungs is higher for our device than for the TOBI Podhaler®. The doses will be administered in a rising order to be able to react to possible adverse events and to possibly end the study prematurely for the participant if necessary. During this study DP Tobramycin will only be administered in a hospital setting, so in case of an emergency adequate action can be taken (e.g. salbutamol for bronchoconstriction).

DP Tobramycin contains only tobramycin and lactose, both compounds that are approved for pulmonary administration. Furthermore, tobramycin has been used for long-term therapy for decades already. The long term risks for tobramycin, predominantly nephrotoxicity and ototoxicity, are due to prolonged exposure to and cumulation of the compound in these organs. In this study, a patient receives four individual doses in four weeks, and no chronic therapy. To reduce the risk of interaction with other drugs, we will screen for cyclosporin, cisplatin, amfotericin B, cephalosporins, polymyxins, vancomycin and NSAIDs. Use of these products should be ceased if possible (NSAIDs), otherwise the patient cannot participate in the study. Therefore, we conclude that the long term risks are negligible.

13. REFERENCES

1. Barker AF, Couch L, Fiel SB, et al. Tobramycin solution for inhalation reduces sputum pseudomonas aeruginosa density in bronchiectasis. *Am J Respir Crit Care Med*. 2000;162(2 Pt 1):481-485.

2. Goeminne P, Dupont L. Non-cystic fibrosis bronchiectasis: Diagnosis and management in 21st century. *Postgrad Med J*. 2010;86(1018):493-501.

3. Pappalettera M, Aliberti S, Castellotti P, Ruvolo L, Giunta V, Blasi F. Bronchiectasis: An update. *Clin Respir J*. 2009;3(3):126-134.

4. Pasteur MC, Bilton D, Hill AT, British Thoracic Society Bronchiectasis non-CF Guideline Group. British thoracic society guideline for non-CF bronchiectasis. *Thorax*. 2010;65 Suppl 1:i1-58.

5. Angrill J, Agusti C, De Celis R, et al. Bronchial inflammation and colonization in patients with clinically stable bronchiectasis. *Am J Respir Crit Care Med*. 2001;164(9):1628-1632.

6. Angrill J, Agusti C, de Celis R, et al. Bacterial colonisation in patients with bronchiectasis: Microbiological pattern and risk factors. *Thorax*. 2002;57(1):15-19.

7. Nicotra MB, Rivera M, Dale AM, Shepherd R, Carter R. Clinical, pathophysiologic, and microbiologic characterization of bronchiectasis in an aging cohort. *Chest*. 1995;108(4):955-961.

8. Miszkiel KA, Wells AU, Rubens MB, Cole PJ, Hansell DM. Effects of airway infection by pseudomonas aeruginosa: A computed tomographic study. *Thorax*. 1997;52(3):260-264.

9. Ho PL, Chan KN, Ip MS, et al. The effect of pseudomonas aeruginosa infection on clinical parameters in steady-state bronchiectasis. *Chest*. 1998;114(6):1594-1598.

10. Lynch DA, Newell J, Hale V, et al. Correlation of CT findings with clinical evaluations in 261 patients with symptomatic bronchiectasis. *AJR Am J Roentgenol*. 1999;173(1):53-58.

11. Wilson CB, Jones PW, O'Leary CJ, Hansell DM, Cole PJ, Wilson R. Effect of sputum bacteriology on the quality of life of patients with bronchiectasis. *Eur Respir J*. 1997;10(8):1754-1760.

12. Westerman EM, De Boer AH, Le Brun PP, et al. Dry powder inhalation of colistin in cystic fibrosis patients: A single dose pilot study. *J Cyst Fibros*. 2007;6(4):284-292.

13. Geller DE, Konstan MW, Smith J, Noonberg SB, Conrad C. Novel tobramycin inhalation powder in cystic fibrosis subjects: Pharmacokinetics and safety. *Pediatr Pulmonol*. 2007;42(4):307-313.

14. Konstan MW, Geller DE, Minic P, Brockhaus F, Zhang J, Angyalosi G. Tobramycin inhalation powder for P. aeruginosa infection in cystic fibrosis: The EVOLVE trial. *Pediatr Pulmonol*. 2010.

15. Konstan MW, Flume PA, Kappler M, et al. Safety, efficacy and convenience of tobramycin inhalation powder in cystic fibrosis patients: The EAGER trial. *J Cyst Fibros*. 2011;10(1):54-61.

16. Le Brun PP, de Boer AH, Mannes GP, et al. Dry powder inhalation of antibiotics in cystic fibrosis therapy: Part 2. inhalation of a novel colistin dry powder formulation: A feasibility study in healthy volunteers and patients. *Eur J Pharm Biopharm*. 2002;54(1):25-32.

17. Newhouse MT, Hirst PH, Duddu SP, et al. Inhalation of a dry powder tobramycin PulmoSphere formulation in healthy volunteers. *Chest*. 2003;124(1):360-366.

18. Pilcer G, Goole J, Van Gansbeke B, et al. Pharmacoscintigraphic and pharmacokinetic evaluation of tobramycin DPI formulations in cystic fibrosis patients. *Eur J Pharm Biopharm*. 2008;68(2):413-421.

19. Ramsey BW, Dorkin HL, Eisenberg JD, et al. Efficacy of aerosolized tobramycin in patients with cystic fibrosis. *N Engl J Med*. 1993;328(24):1740-1746.

20. O'Donnell AE, Barker AF, Ilowite JS, Fick RB. Treatment of idiopathic bronchiectasis with aerosolized recombinant human DNase I. rhDNase study group. *Chest*. 1998;113(5):1329-1334.

21. Orriols R, Roig J, Ferrer J, et al. Inhaled antibiotic therapy in non-cystic fibrosis patients with bronchiectasis and chronic bronchial infection by pseudomonas aeruginosa. *Respir Med*. 1999;93(7):476-480.

22. Couch LA. Treatment with tobramycin solution for inhalation in bronchiectasis patients with pseudomonas aeruginosa. *Chest*. 2001;120(3 Suppl):114S-117S.

23. Proost JH, Meijer DK. MW/Pharm, an integrated software package for drug dosage regimen calculation and therapeutic drug monitoring. *Comput Biol Med*. 1992;22(3):155-163.

24. Vakulenko SB, Mobashery S. Versatility of aminoglycosides and prospects for their future. *Clin Microbiol Rev*. 2003;16(3):430-450.

25. MacLusky IB, Gold R, Corey M, Levison H. Long-term effects of inhaled tobramycin in patients with cystic fibrosis colonized with pseudomonas aeruginosa. *Pediatr Pulmonol*. 1989;7(1):42-48.

26. Steinkamp G, Tummler B, Gappa M, et al. Long-term tobramycin aerosol therapy in cystic fibrosis. *Pediatr Pulmonol*. 1989;6(2):91-98.

27. Smith AL, Ramsey BW, Hedges DL, et al. Safety of aerosol tobramycin administration for 3 months to patients with cystic fibrosis. *Pediatr Pulmonol*. 1989;7(4):265-271.
